# Restaurant Inspection Scores and Foodborne Disease

**DOI:** 10.3201/eid1004.030343

**Published:** 2004-04

**Authors:** Timothy F. Jones, Boris I. Pavlin, Bonnie J. LaFleur, L. Amanda Ingram, William Schaffner

**Affiliations:** * Tennessee Department of Health, Nashville, Tennessee, USA; † Vanderbilt University School of Medicine, Nashville, Tennessee, USA

**Keywords:** Restaurant, inspection, food safety, foodborne disease

## Abstract

Restaurants in the United States are regularly inspected by health departments, but few data exist regarding the effect of restaurant inspections on food safety. We examined statewide inspection records from January 1993 through April 2000. Data were available from 167,574 restaurant inspections. From 1993 to 2000, mean scores rose steadily from 80.2 to 83.8. Mean inspection scores of individual inspectors were 69–92. None of the 12 most commonly cited violations were critical food safety hazards. Establishments scoring <60 had a mean improvement of 16 points on subsequent inspections. Mean scores of restaurants experiencing foodborne disease outbreaks did not differ from restaurants with no reported outbreaks. A variety of factors influence the uniformity of restaurant inspections. The restaurant inspection system should be examined to identify ways to ensure food safety.

More than 54 billion meals are served at 844,000 commercial food establishments in the United States each year (*1*); 46% of the money Americans spend on food goes for restaurant meals (*2*). On a typical day, 44% of adults in the United States eat at a restaurant (*1*). Of a mean 550 foodborne disease outbreaks reported to the Centers for Disease Control and Prevention each year from 1993 through 1997, >40% were attributed to commercial food establishments (*3*). Preventing restaurant-associated foodborne disease outbreaks is an important task of public health departments.

Restaurants in the United States are regularly inspected by local, county, or state health department personnel. The guidelines of the U.S. Food and Drug Administration state that “a principal goal to be achieved by a food establishment inspection is to prevent foodborne disease” (*4*). Although restaurant inspections are one of a number of measures intended to enhance food safety, they are a highly visible responsibility of local health departments. In many parts of the country, restaurant inspection scores are easily accessible to the public through the Internet or are disseminated through local news media. We postulated that an inspection system that effectively addressed the goal of improving food safety would be uniform, consistent, and focused on identifying characteristics known to affect food safety. We examined data on restaurant inspections in the state of Tennessee to determine whether the system there demonstrated such characteristics.

## Methods

Statewide restaurant inspection data from Tennessee from January 1993 through April 2000 were analyzed. Semiannual inspections were required of all restaurants with permits for preparing and serving food; all routine inspections during this period were included in the analysis. Special inspections performed in response to customer complaints or to follow-up on deficiencies noted in semiannual inspections were not included. We did not include inspections of schools, correctional facilities, and bars that did not serve food. Inspections were performed by state health department employees, or by county health department employees in most metropolitan areas of the state, in accordance with uniform state laws and regulations. All inspectors undergo uniform training and certification by state health department management personnel. To avoid skewing results by including persons performing very few inspections per year, when comparing mean inspection scores by inspector, we included those performing at least 100 inspections during the study period.

Inspections were performed by using standardized forms including 44 scored items with a possible total score of 100. Of those 44 items, 13 were designated as “critical” (Appendix). Critical items are violations “which are more likely to contribute to food contamination, illness, or environmental degradation and represent substantial public health hazards and [are] most closely associated with potential foodborne disease transmission” (*4*). Data available for each inspection included overall score, specific violations cited, establishment name and identification number, county, date of inspection, inspector, and time spent on inspection.

For comparison purposes, a convenience sample of 19,700 inspections of 2,379 restaurants known to serve distinct types of international or regional cuisine were analyzed. In addition, a convenience sample of 46,700 inspections of 5,300 restaurants were compared on the basis of type of table service. These restaurants were selected based on being well-known to investigators as to type of service or cuisine. Fast-food restaurants were defined as establishments where food was paid for before eating. Full-service restaurants were defined as establishments where patrons were served at tables and meals were paid for after consumption. Establishments that were difficult to classify or not known to investigators were not included. Data were entered in a centrally maintained database and were analyzed with Excel (Microsoft, Redmond, WA), SAS 8.0 (SAS, Cary, NC), and EpiInfo 6.2 software (*5*).

## Results

**Figure 1:**
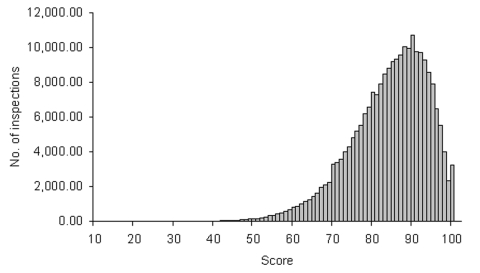
Distribution of scores of restaurants inspected statewide from July 1993 to June 2000, based on a standardized inspection with 44 scored items and a maximum score of 100.

**Figure 2:**
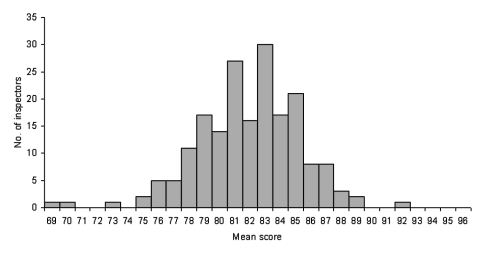
Mean score of all restaurants inspected by each inspector, for inspectors performing at least 100 inspections during the study period.

All commercial establishments preparing or serving food in Tennessee are required to hold a permit from the Tennessee Department of Health. Tennessee has approximately 13,000 restaurants licensed and approximately 145 restaurant inspectors. Data were available from 167,574 restaurant inspections, involving 29,008 unique restaurants and 248 inspectors during the study period. During this period, individual restaurant scores were 13–100; the mean was 82.2, and the median was 83 (Figure 1). Among 190 inspectors performing at least 100 inspections during the study period, mean inspection scores of individual inspectors were 69–92, with a median of 82 (Figure 2). Mean scores of restaurants within each of the 95 counties in Tennessee were 75–88. From 1993 to 2000, the mean inspection score rose steadily from 80.2 to 83.8, and the mean number of violations cited per inspection fell from 11.1 to 9.9.

During routine restaurant inspections, the most commonly cited violations were for unclean surfaces of equipment that did not contact food and floors or walls appearing unclean, poorly constructed, or in poor repair (Table). None of the 12 most commonly cited violations were among those designated as “critical” food safety hazards. The critical violation most commonly cited was the improper storage or use of toxic items (for example, storing cleaning fluids on a shelf next to food), which was the 13th most commonly cited violation during routine inspections.

**Table:** Number of times each of the 15 most common violations were cited on routine restaurant inspections statewide, 1993–2000

Violation	Frequency
Nonfood contact surfaces of equipment and utensils clean	142,924
Floors constructed, drained, clean, good repair, covering, installation, dustless cleaning methods	142,812
Walls, ceilings, attached equipment, constructed, good repair, clean surfaces, dustless cleaning methods	136,178
Food-contact surfaces of equipment and utensils clean, free of abrasives, detergents	127,156
Non-food contact surfaces designed, constructed, maintained, installed, located	111,813
Food protection during storage, preparation, display, service, transportation	101,126
Food (ice) contact surfaces designed, constructed, maintained, installed	96,657
Premises maintained free of litter, unnecessary articles, cleaning maintenance equipment properly stored	91,422
Toilet rooms enclosed, self-closing doors, fixtures good repair, clean, hand-cleanser, sanitary towels, hand-drying devices provided, proper waste receptacles	88,140
Single-service articles, storage, dispensing	81,562
Containers or receptacles, covered, adequate number, insect and rodent proof, frequency, clean	78,143
Lighting provided as required, fixtures shielded	71,453
Toxics items properly stored, labeled, used^a^	70,995
Thermometers provided and conspicuous	69,595
Food protection during storage, preparation, display, service, transportation	69,059

^a^“Critical” item.

Among restaurant inspections with a total score of >80, at lease one critical violation was cited in 44% of those inspections (mean number of critical violations was 0.6, mean number of noncritical violations was 6.3). A critical violation was cited in 9,127 inspections with a final score >90. Among inspections with scores of 60 to 80, a mean of 2.4 critical and 11.4 noncritical violations were cited; for inspections with a score <60, the means were 5.4 and 16, respectively. In 1,698 inspections with a score of 60 to 80, no critical violations were cited.

**Figure 3:**
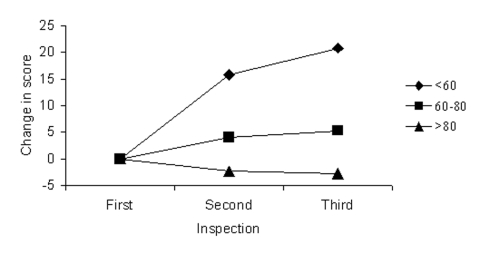
Mean change in scores in subsequent two inspections, for restaurants with an initial score on routine inspection of <60, 60–80, or >80.

During this period, restaurants with a score >60 tended to have fairly stable scores on subsequent inspections, with a mean drop of 2 points on the subsequent inspection (Figure 3). Establishments scoring <60 had a mean improvement of 16 points on the subsequent routine inspection, with an additional mean increase of 5 on the next inspection.

Restaurant inspection data were available from 49 restaurants that were identified as the source of foodborne disease outbreaks investigated by health departments in Tennessee from 1999 to 2002. The mean score of the last routine inspection before the reported outbreak was 81.2, and the mean score of the inspection previous to the most recent inspection was 81.6. These scores do not differ significantly from the mean scores of all restaurant inspections during the study period. The rank order of most commonly cited critical violations on routine inspections of restaurants subsequently involved in outbreaks was similar to restaurants not involved in outbreaks. While the two most common critical violations (proper storage of toxic items and good handwashing and hygienic practices) were more likely to have been cited during the two routine inspections before an outbreak occurred at a restaurant, the number of reported outbreaks is small, and the conclusions that can be drawn from this observation are limited.

Under state law, restaurants in Tennessee are inspected once every 6 months. The median time between successive inspections during this period was 175 days; 88% of inspections were performed from 90 to 270 days after the previous inspection. Mean scores were similar in restaurants inspected less than or more than 180 days since the previous inspection (81.7 and 82.7, respectively) and in restaurants inspected within 200 days compared to >270 days since the previous inspection (81.9 and 83.7, respectively).

Fast-food restaurants (mean score = 79.9) had mean scores similar to independent (80.9) or chain (82.1) full-service restaurants. Small variations were noted in mean scores of restaurants serving specific types of cuisine, such as Thai (83.1), barbeque (82.9), pizza (82.3), Italian (81.0), Chinese (77.7), Mexican (77.4), Japanese (76.4), and Indian (74.8) foods.

## Discussion

These data demonstrate that, during a 7-year period in Tennessee, routine restaurant inspection scores varied substantially over time, by region, and by person performing the inspection. While regional variations in the general quality of food service establishments are possible, this factor is unlikely to account for a substantial proportion of the observed differences. Restaurant inspections performed by a single observer are difficult to standardize and easily influenced by subjective interpretation. Further analyses can be performed that examine the variation in scores on the basis of such things as demographic characteristics of inspectors and time since last standardized training; these analyses can also be done prospective studies of interobserver variability at the same establishments.

All restaurant inspections in Tennessee during this period were performed under the same laws and procedures and using standard data collection forms. New inspectors undergo standardized training before performing inspections alone, though during this study period no mechanism for formal periodic restandardization after initial training existed. Since this study period (and independently of this study) the health department has instituted statewide retraining of all inspectors, regardless of length of experience. Whether periodic standardized retraining affects the variables assessed in this study is yet to be determined.

Despite the ubiquity of restaurant inspections, few studies have been published about the correlations between restaurant inspection scores or violations and foodborne illness, and the conclusions are conflicting (*6*–*12*). Methodologic problems, including the rarity of reported foodborne outbreaks in relation to the number of restaurants and the small percentage of suspected foodborne illnesses linked to epidemiologically confirmed, restaurant-associated outbreaks, make such analyses difficult. The intensity of surveillance for foodborne disease can markedly influence the number of foodborne disease outbreaks reported in a jurisdiction, and a substantial proportion of restaurant-associated foodborne illnesses probably goes unreported. This study did not assess foodborne illness as an endpoint but rather examined characteristics of an inspection system that would be expected to be associated with a consistent, predictable, and reliable foodborne illness prevention system. The limited data available on outbreaks in Tennessee suggest that restaurant inspection scores alone do not predict the likelihood of a foodborne outbreak occurring in a particular establishment.

We are not aware of published data addressing which items on a routine restaurant inspection are demonstrated to lead to improved food safety within an establishment. The Tennessee Department of Health inspection protocol and the federal Food Code (*4*) after which it is modeled include assessment of a variety of factors of limited importance in directly preventing foodborne illness. These items include condition surfaces that do not contact food, floors, walls and ceilings, lighting, and ventilation. Such factors would be expected to substantially influence an observer’s impression of overall cleanliness and safety of an operation, but isolated characteristics have not been shown to correlate with food safety. A substantial number of inspections with a final score of >90 also had critical violations; likewise, some restaurants with scores <80 had no critical violations. While most common violations are noncritical items, these data serve as a reminder that overall score alone is not necessarily a sufficient measure of restaurant safety. A number of studies have examined the effect of inspection frequency on restaurant sanitation (*9*,*13*–*16*). We did not observe a meaningful difference in scores on the basis of time since previous inspection, although because of state laws requiring inspections every 6 months, the variation in intervals was limited. Data from other programs with more variation in inspection frequency might be helpful in assessing the potential effect of time since last inspection.

Restaurant inspections serve an additional goal of ensuring immediate physical safety of patrons and workers in the environment. Further studies to determine the most efficient and effective methods for assessing factors associated with food safety will be important to help improve the inspection system. Recent introduction of Hazard Analysis and Critical Control Points systems in many areas of the foodservice industry are an attempt to focus proactively on issues important to food safety (*4*).

Given the universal performance of restaurant inspections in the United States, no large group of identical restaurants under similar social conditions exist to compare as “controls” to assess the direct effects of inspections. Simply the anticipation of routine inspections probably improves compliance with food safety guidelines and laws (*17*). The most appropriate mechanism for measuring restaurant sanitation and sharing the results remains a subject of much debate (*18*–*21*). Recent regular dissemination of local restaurant scores in print and broadcast media in Tennessee may have increased establishments’ attention to addressing deficits. Many businesses may improve compliance with regulations to avoid bad publicity and negative economic repercussions. While no studies have been done to show that these types of negative reproductions have led to decreased foodborne illness in Tennessee or elsewhere, the restaurant inspection system may be an effective mechanism to motivate change within the industry.

Public perception about the relative cleanliness or safety of particular types of restaurants may not reflect reality. Many voluntary interventions, such as strict corporate policies on establishment design, equipment, and hygiene within a particular company can affect a large number of restaurants over a wide geographic area. Such policies and procedures within large multistate corporations are unlikely to be substantially affected by local inspection policies. In contrast, restaurants serving specific ethnic or otherwise easily categorized cuisines are more likely to be locally owned and operated and may be more influenced by local management policies. More systematic assessment of this issue will help focus preventive intervention efforts.

This study suggests that a variety of factors influence the uniformity and reliability of routine restaurant inspections in preventing foodborne disease. Some of these factors might be modified by policies designed to ensure periodic retraining and systematic standardization among inspection evaluations within a jurisdiction. Further evaluating factors important in food safety and how best to control them will be important in improving the system. The Centers for Disease Control and Prevention, in collaboration with the Food and Drug Administration and other agencies, has recently launched an Environmental Health Specialist Network project in seven states. This program will systematically address issues of restaurant inspections and their relationship to food safety and might contribute to our understanding of this system and efforts to improve it.

## Appendix. “Critical” items on the Tennessee Department of Health food service establishment inspection report^a^

1. Food is from an approved source in sound condition, with no spoilage.

2. Potentially hazardous food meets temperature requirements during storage, preparation, display, service and transportation.

3. Facilities are available to maintain product temperature.

4. Unwrapped and potentially hazardous food is not reserved.

5. Personnel with infections are restricted from potentially hazardous work.

6. Hands are washed according to good hygienic practices.

7. Food equipment and utensils are sanitized using appropriate methods.

8. Water comes from a safe source, with hot and cold water under appropriate pressure.

9. Sewage and waste water disposal are sanitary.

10. Plumbing prevents backflow, back-siphonage or dangerous cross-connections.

11. Toilet and handwashing facilities are convenient, accessible, well-designed, and appropriately installed.

12. There is no detectable presence of insects, rodents, birds, turtles, or other animals and outer openings are protected.

13. Toxic items are properly stored, labeled, and used.

^a^Critical items are violations “which are more likely to contribute to food contamination, illness, or environmental degradation and represent substantial public health hazards and [are] most closely associated with potential foodborne disease transmission” (4).
